# Stable Water Use Efficiency of Tibetan Alpine Meadows in Past Half Century: Evidence from Wool δ^13^C Values

**DOI:** 10.1371/journal.pone.0144752

**Published:** 2015-12-11

**Authors:** Hao Yang, Nianpeng He, Yongtao He, Shenggong Li, Peili Shi, Xianzhou Zhang

**Affiliations:** Key Laboratory of Ecosystem Observation and Modeling, Institute of Geographic Sciences and Natural Resources Research, Chinese Academy of Sciences, Beijing 100101, China; University of Leipzig, GERMANY

## Abstract

Understanding the influences of climatic changes on water use efficiency (WUE) of Tibetan alpine meadows is important for predicting their long-term net primary productivity (NPP) because they are considered very sensitive to climate change. Here, we collected wool materials produced from 1962 to 2010 and investigated the long-term WUE of an alpine meadow in Tibet on basis of the carbon isotope values of vegetation (*δ* ^13^C_veg_). The values of *δ* ^13^C_veg_ decreased by 1.34‰ during 1962–2010, similar to changes in *δ* ^13^C values of atmospheric CO_2_. Carbon isotope discrimination was highly variable and no trend was apparent in the past half century. Intrinsic water use efficiency (*W*_*i*_) increased by 18 μmol·mol^–1^ (approximately 23.5%) during 1962–2010 because the increase in the intercellular CO_2_ concentration (46 μmol·mol^–1^) was less than that in the atmospheric CO_2_ concentration (*C*_*a*_, 73 μmol·mol^–1^). In addition, *W*_*i*_ increased significantly with increasing growing season temperature and *C*_*a*_. However, effective water use efficiency (*W*_*e*_) remained relatively stable, because of increasing vapor pressure deficit. *C*_*a*_, precipitation, and growing season temperature collectively explained 45% of the variation of *W*_*e*_. Our findings indicate that the *W*_e_ of alpine meadows in the Tibetan Plateau remained relatively stable by physiological adjustment to elevated *C*_*a*_ and growing season temperature. These findings improve our understanding and the capacity to predict NPP of these ecosystems under global change scenarios.

## Introduction

The Tibetan Plateau, referred to as the Earth’s “third pole,” is highly sensitive to climate change, and climate warming has been widely observed here during the past several decades [1–2]. The mean annual temperature (MAT) increased from 1961 to 2010, accompanied by a strong increase in atmospheric CO_2_ concentration (*C*_a_) and a slight decrease in photosynthetically active radiation, although the mean annual precipitation (MAP) did not vary apparently during this period [2–3]. The way in which these changes in climate and *C*_a_ influence the net primary productivity (NPP) of alpine meadows is of great concern [2]. The NPP of grasslands generally increases with increasing promotion of plant growth by precipitation or increased water use efficiency (WUE) related to the leaf area index [4]. The observed increases in growing season temperature (*GST*) and *C*_a_ in the Tibetan Plateau may increase Rubisco enzyme activity, stimulate leaf photosynthesis, enhance NPP, and result in higher WUE [5–6]. Here, we assumed that the WUE of alpine meadows would increase under scenarios of warming and increasing *C*_a_ and result in higher NPP in the Tibetan Plateau as shown by Piao and others [2].

Various methods have been used to evaluate WUE of vegetation, including: 1) estimation from measurements of photosynthetic parameters [7]; 2) indirect estimation from the carbon isotope composition (*δ* ^13^C) of leaves [8]; 3) calculation from measurements of vegetation biomass and the biomass/precipitation index [9]; and 4) calculation from eddy covariance measurements of CO_2_ and H_2_O fluxes [4]. Photosynthesis- and *δ* ^13^C-based methods are generally used at the species or individual plant levels; biomass- and eddy covariance-based methods are performed for ecosystem-level assessments. Because of limitations related to plant lifespan, sampling, and measurement techniques, reports on long-term ecosystem-level WUE of grassland are limited. In the Tibet Plateau, most previous studies on WUE or *δ* ^13^C values focused on the species or individual plant levels. For example, the WUE of perennials were found to be higher than that of annuals in terms in individual plants as shown by plant carbon isotope measurements [10]. At the ecosystem level the WUE of alpine meadow is higher at the middle of the growing season and low at the beginning and end of the growing season during a year, and it is higher in a wet year than in a normal year as revealed by eddy covariance [4, 11]. However, it is not clear how the WUE of grasslands changes over longer time scales at the ecosystem level.

New approaches using *δ* ^13^C values of animal tissues such as horns have been successfully used and the stable long-term and ecosystem-level WUE in the Alps alpine grassland was previously reported [12]; the isotopic composition of these tissues can reflect spatiotemporal information about vegetation in grazed areas. An apparent ^13^C enrichment of wool related to the animal’s diet, called the carbon isotopic “diet–wool shift” (*ε*) [13–15], results from ^13^C fractionation during digestion or metabolism [16]. The ^13^C discrimination of vegetation (^*13*^ *Δ*_*veg*_) can be calculated, and long-term and ecosystem-level WUE can be deduced based on *ε*, the *δ* ^13^C values of animal tissues, and the *δ* ^13^C value of atmospheric CO_2_ (*δ* ^13^C_air_).

On the Tibetan Plateau, wool-based products such as fur coats and rugs have been preserved by farmers and monks, and these materials provide an opportunity to assess samples produced in different years. In this study, we collected samples of wool products produced from 1962 to 2010 in a natural alpine meadow region of the Tibetan Plateau and analyzed the *δ* ^13^C values of the samples. Our objectives were to 1) assess long-term changes in WUE of alpine meadows at the ecosystem level, 2) explore the underlying drivers of WUE, and 3) examine the hypothesis that WUE of alpine meadows increases and may result in higher NPP on the Tibetan Plateau under a warming climate with increasing *C*_a_. Our findings can improve the capacity to predict NPP of alpine meadows under global change scenarios.

## Materials and Methods

### Study area

**Fig 1:**
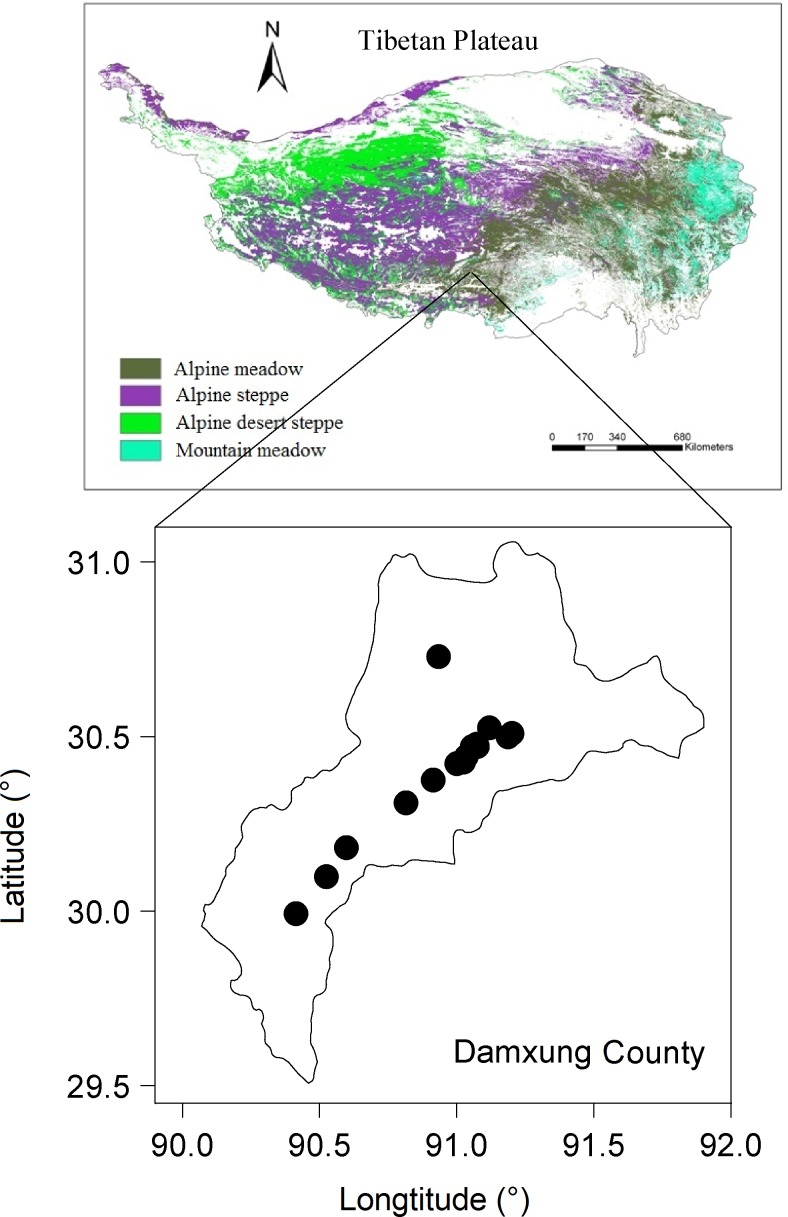
Sampling sites in Damxung County, Tibetan Plateau. This figure is a modification from the Vegetation Map of The People’s Republic of China (1:1000000) [38].

The study area, a natural alpine meadow on the southern Tibetan Plateau, is located in Damxung County (90°45′–91°31′E, 29°31′–30°25′N, 10036 km^–2^ including 30% grassland area) in the Xizang Autonomous Region, People’s Republic of China (Fig 1). Damxung County has a mean elevation of 4200 m and a plateau monsoon climate. MAT is approximately 1.3°C, and MAP is 477 mm, the majority of which (90%) falls during the growing season (June–September). The dominant plant species in the meadow are *Stipa capillacea*, *Carex montis-everestii*, and *Kobresia pygmaea*, together accounting for 69% of total aboveground biomass (measured during 2009–2012 by the Damxung Grassland Research Station [91°05′E, 30°25′N], Chinese Academy of Sciences). Twenty-one plant species were observed during 2009–2012, and all are C_3_ species. The study area is freely grazed by sheep and yak, along with a low number of goats; the mean number of livestock in 1985–2010 was 0.55 ± 0.02 million on an area of approximately 0.3 million ha, which suggests that the grazing pressure was low and varied little. Winter forage consists mainly of dry grasses that are harvested locally in August.

### Sampling

In September 2011, 15 villages in Damxung County (4230–4300 m a.s.l.) were selected randomly as sampling sites (Fig 1). Wool materials from local sheep, including fur coats, rugs, and recently sheared wool, were obtained from farmers and monks. Wool samples were dated by asking farmers and monks about the age of the samples. The sample size for each year varied from 1 to 12 depending on how many samples we were able to collect. No specific permission was required for these sampling sites and activities because the temples were open and sampling was permitted by monks and farmers. The wool was only produced by sheep, not endangered or protected species. In total, 106 wool samples dating from 1962 to 2010 were collected. Three assumptions are made for deriving temporal information about vegetation from these samples [12]: that (i) sheep grazed on the same meadows every year, (ii) the dietary preferences of sheep did not change over time, and (iii) the value of *ε* was stable among individual sheep.

### Sample preparation and carbon isotope analysis

The wool samples were cleaned following the procedure of Schwertl and others [17], and samples (0.2–0.4 mg) were packed into tin cups for isotope analysis. The *δ* ^13^C values of wool (*δ* ^13^C_wool_) were measured using an elemental analyzer (NA 1110; Carlo Erba, Milan) interfaced (ConFlo III; Finnigan MAT, Bremen) with an isotope ratio mass spectrometer (Delta Plus; Finnigan MAT). The *δ* ^13^C_wool_ values were specified as *δ* ^13^C relative to the Vienna Pee Dee Belemnite (VPDB) standard:

$$\delta^{13}C_{\mathrm{wool}}=\frac{R_{\mathrm{sample}}}{R_{\mathrm{standard}}}-1$$ (1)

where *R*_sample_ and *R*_standard_ are the ratios of ^13^C/^12^C in the samples and standard, respectively. Each sample was measured against a laboratory working CO_2_ standard, which was previously calibrated against an International Atomic Energy Agency secondary standard (IAEA-CH6, calibration accuracy of 0.06‰ SD). After every tenth sample, a solid internal lab standard (SILS) with a C/N ratio similar to that of the sample material was run as a blind control. The SILSs were previously calibrated against IAEA-CH6. The precision of the repeated sample was 0.11‰.

### Estimating vegetation *δ* ^13^C

The values of *ε*, the vegetation to wool fractionation, are approximately 2–4‰ in C_3_ or C_3_/C_4_ mixed vegetation [15, 17, 18, 19] and are independent of altitude [13]. Based on a previous study by Maennel and others [13] from a region with similar background (C_3_ plants grazed by sheep, high altitude), 3.2‰ was used as *ε*. Hence, *δ* ^13^C_veg_ was estimated as

$$\delta^{13}C_{\mathrm{veg}}=\delta^{13}C_{\mathrm{wool}}-3.2‰$$ (2)

### Estimating WUE

The ^*13*^ *Δ*_veg_ is derived from *δ* ^13^C_veg_ and *δ* ^13^C_air_:

Δ13veg=δ13Cair−δ13Cveg1+δ13Cveg/1000 (3)

The ^*13*^ *Δ*_*veg*_ value captures the main drivers of photosynthetic carbon isotope fractionation [20]. Farquhar and others [8] reported that ^*13*^ *Δ*_*veg*_ depends on the relationship between the photosynthetic carbon assimilation rate (*A*) and stomatal conductance (*g*_*s*_), which determines the ratio of intercellular to atmospheric CO_2_ concentrations (*C*_*i*_ */C*_*a*_) in C_3_ plants:

$$\frac{C_{i}}{C_{a}}=\frac{{13_{\Delta }}_{\mathrm{veg}}-a}{b-a}$$ (4)

where *a* is the discrimination of ^13^C during the diffusion of CO_2_ through stomata (4.4‰) and *b* is the net fractionation by carboxylation (27‰).

Hence, intrinsic water use efficiency (*W*_*i*_) can be calculated as follows:

$$W_{i}=\frac{A}{g_{s}}=\frac{C_{a}-C_{i}}{1.6}=\frac{C_{a}(1-\frac{C_{i}}{C_{a}})}{1.6}$$ (5)

where *g*_*s*_ is leaf stomatal conductance of water vapor and 1.6 is the ratio of the diffusivity of water vapor and *C*_*a*_. *W*_*i*_ is regarded as the potential WUE assuming a constant evaporative demand and is used to assess long-term trends in the balance between carbon gain and intrinsic water loss of plants. Under variable environment conditions, effective WUE (*W*_*e*_) is used as the actual WUE because it considers the effect of the water vapor pressure concentration gradient between intercellular spaces and the atmosphere (*v*) [8]:

$$W_{e}=\frac{A}{E}=\frac{C_{a}-C_{i}}{1.6v}=\frac{C_{a}(1-\frac{C_{i}}{C_{a}})}{1.6v}$$ (6)

where *E* is the leaf transpiration rate.

### Climate data, *C*_*a*_, and *δ* ^*13*^ *Cair*

Climate data for Damxung station were obtained from the China Meteorological Administration (CMA). *C*_*a*_ and *δ* ^*13*^ *C*_air_ were estimated following Wittmer and others [21] and Barbosa and others [12] and were required for the calculation of ^*13*^ *Δ*_veg_ (Eqs 3 and 4) and WUE (Eqs 5 and 6). The *δ* ^*13*^ *C*_air_ values for 1991–2010 were obtained from the US National Oceanic and Atmospheric Administration using data from the Waliguan station (100°54′E, 36°17′N, 3810 m a.s.l.), the closest meteorological station on the Tibetan Plateau.

$$C_{a}=16081\times t^{2}-62345\times t+60735$$ (7)

where *t* is the sampling year/1000. The root mean squared error for the overall *C*_*a*_ model was 1.4 μmol·mol^–1^. A cubic function was fitted to *δ* ^13^C_air_ to estimate mean annual values. The model was:

$$\delta^{13}C_{\mathrm{air}}=13675.5085\times t^{3}-81341.3526\times t^{2}+161233.8290\times t-106514.4913$$ (8)

where *t* is the sampling year/1000. The root mean squared error for the overall *δ* ^13^C_air_ was 0.08‰. To calculate mean growing-season *δ* ^13^C_air_, a seasonal correction factor of 0.14‰ was added because the growing-season mean is 0.14‰ greater than the annual mean [21].

### Estimation of vapor pressure concentration gradient (*v*)

The value of *v* was estimated by vapor pressure deficit (VPD) and was used to calculate *W*_*e*_ (Eq 6), with the assumption that leaf and air temperature were the same. The saturation vapor pressure (*e*) was related to air temperature (*T*) and was obtained as follows [22]:

$$e(T)={0.6108e}^{\left(\frac{17.27T}{T+237.3}\right)}$$ (9)

Daily mean saturation vapor pressure (*e*_*s*_) was calculated as

$$e_{s}=\frac{e(T_{max})-e(T_{min})}{2}$$ (10)

Actual vapor pressure (*e*_*a*_) was calculated as

$$e_{a}=e_{s}\times RH$$ (11)

where *RH* is relative humidity. Then, VPD was given by:

$$VPD=e_{s}-e_{a}$$ (12)

Eq 12 was the relative humidity fraction and did not include the decreasing effect of high altitude on total atmospheric pressure (*P*). Thus, we calculated *v* as

$$v=\frac{VPD}{P}$$ (13)

where *P* is total atmospheric pressure (60.6 kPa at 4200 m a.s.l.).

Because plant photosynthesis and transpiration occurred in daytime when sun-light was present, the daytime VPD was more reasonable to use than the VPD here; thus the calculation was performed using the daily VPD as follows.

First, the daily VPD during 1962–2010 were calculated from the climate data (daily) of the CMA using Eqs 9–12. The daily VPD in the growing seasons from July 19, 2003 to August 16, 2010 were validated by the daily VPD calculated using the climate data (hourly) measured by the eddy covariance tower at Damxung Grassland Research Station. The linear regression was:

$$y=0.9924x-0.012,\;{\text{R}}^{2}=0.88,\;\text{n}=883$$ (14)

where *y* was the daily VPD from the eddy covariance tower and *x* was the daily VPD from CMA. Second, the climate data (hourly) measured by the eddy covariance tower and the sunshine time calculated according to the longitude and latitude were used to calculate the relationship between daytime VPD and daily VPD. The linear regression was:

$$y=1.2582x+0.037,\;{\text{R}}^{2}=0.97,\;\text{n}=883$$ (15)

where *y* was the daytime VPD and *x* was the daily VPD. Third, we assumed that Eq 15 was correct during our study period, and the daytime VPD during 1962–2010 was then calculated using Eq 15 and the daily VPD from the CMA.

### Statistical analyses

Because wool is generally shorn in July (the beginning of the growing season), *δ* ^*13*^ *C*_wool_ mainly reflects the isotopic signature of vegetation during the previous growing season. Hence, *C*_*a*_ and *δ* ^*13*^ *C*_air_ values for the previous growing season were used to calculate *C*_*i*_, *W*_*i*_, and *W*_*e*_ and to explore the relationships among them.

All isotope data were tested for normality using the Kolmogorov–Smirnov test and for equality of error variance using Levene’s test. Linear regression was used to explore the changing trends of *W*_*e*_ with time, and the absolute values of the partial correlation coefficient obtained from a partial correlation analysis were used to identify the relative importance of explanatory variables. All statistical analyses were performed using SPSS Version 17.0 (SPSS, Inc., Chicago, IL).

## Results

### Long-term changes in *δ* ^13^C_wool_, *δ* ^13^C_veg_, and ^*13*^ *Δ*_*veg*_

**Fig 2:**
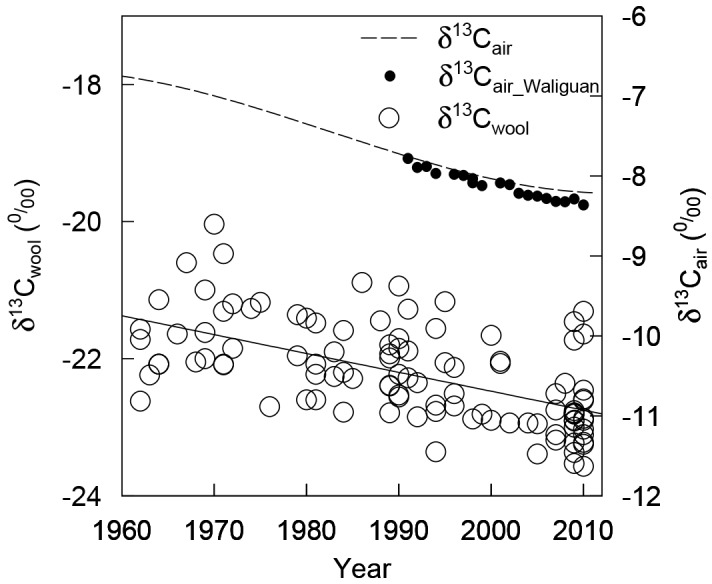
Carbon isotopic composition of wool (*δ* ^13^C_wool_) from Tibetan alpine meadows and of atmospheric CO_2_ (*δ* ^13^C_air,_ *δ* ^13^C_air_Waliguan_). The wool data were fitted with a linear model (y = –0.028x + 32.561, *n* = 106, *P <* 0.001).

**Fig 3:**
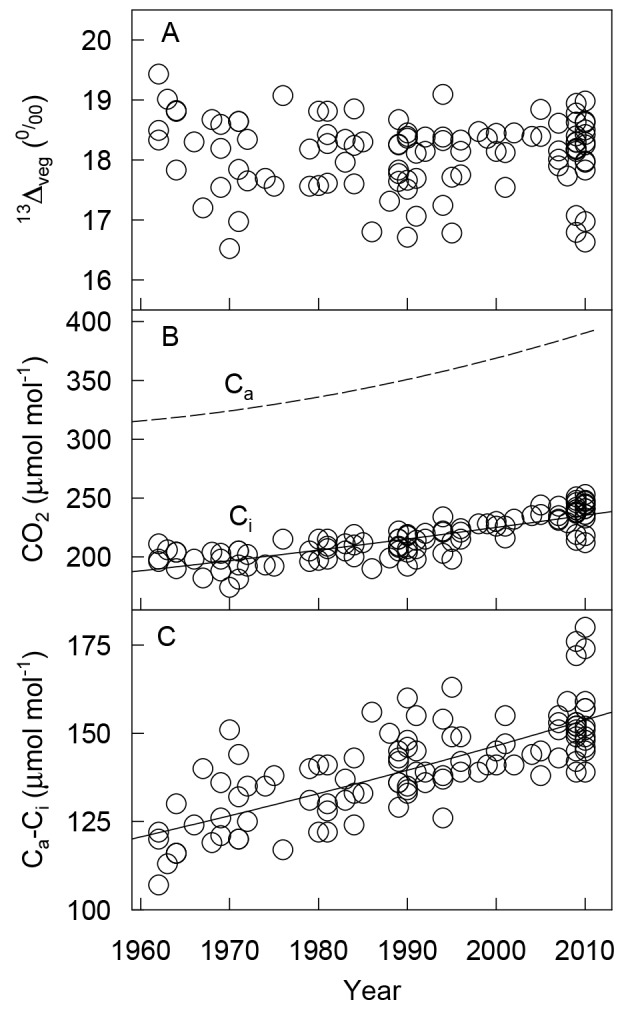
Ecophysiological parameters of Tibetan alpine meadows reconstructed from the *δ* ^13^C time series of wool samples. (A) Carbon isotope discrimination (^*13*^ *Δ*_*veg*_); (B) CO_2_ concentration in the atmosphere (*C*_*a*_, dashed line) and in intercellular space (*C*_*i*_, solid line); (C) the difference between *C*_*a*_ and *C*_*i*_ (*C*_*a*_−*C*_*i*_). The parameter trend lines were calculated using the values derived from *δ* ^13^C of wool samples (*n* = 106). The models fitting *C*_*i*_ and *C*_*a*_−*C*_*i*_ data were y = 0.029e^0.004x^ and y = 0.009e^0.005x^ respectively.

The average value of *δ* ^13^C_wool_ was –22.22‰, ranging from –23.57‰ to –20.04‰. The *δ* ^13^C_wool_ and *δ* ^13^C_veg_ values decreased over time (*R* ^*2*^ = 0.35, *n* = 106, *P* < 0.001) (Fig 2), similar to the trend observed for *δ* ^13^C_air_. Unexpectedly, ^*13*^ *Δ*_*veg*_ was relatively stable over the past half century (Fig 3A) with an average value of 18.10‰ (range, 16.52‰ to 19.43‰).

### Long-term changes in *C*_*i*_, *C*_*i*_/*C*_*a*_, and WUE

**Fig 4:**
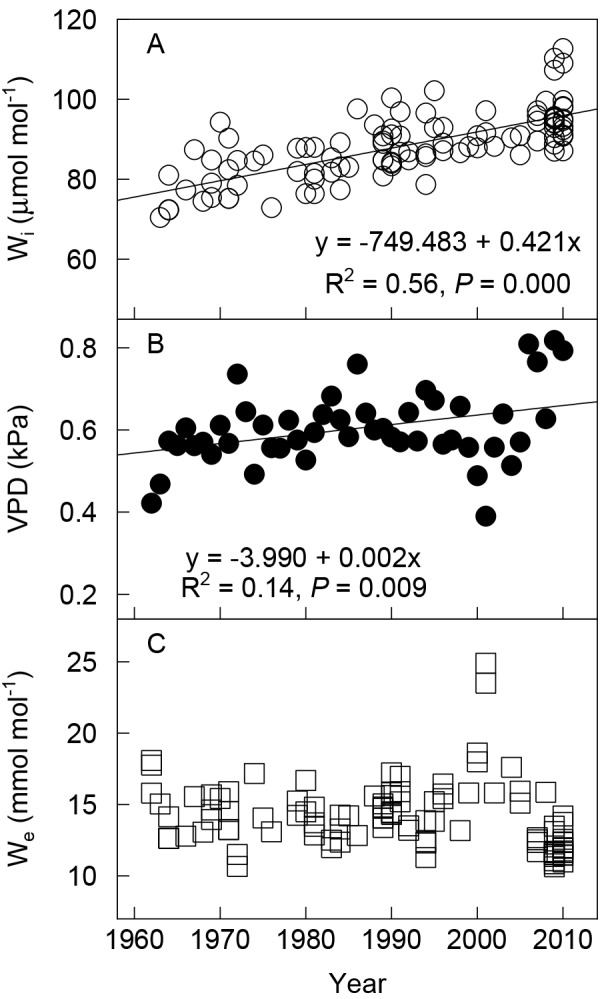
Ecophysiological parameters of Tibetan alpine meadows. (A) Intrinsic water use efficiency (*W*_*i*_), *n* = 106; (B) atmospheric vapor pressure deficit (*VPD*), *n* = 49; (C) effective water use efficiency (*W*_*e*_), *n* = 106. The parameter trend lines were calculated using values derived from *δ* ^13^C of wool samples.

As described by Eq (4) and ^*13*^ *Δ*_*veg*_ values, the *C*_*i*_/*C*_*a*_ ratio changed little during the four decades examined. *C*_*i*_ values increased by 46 μmol·mol^–1^ (*R* ^*2*^ = 0.69, *P* < 0.001) but did not balance the increase in *C*_*a*_ (73 μmol·mol^–1^, Fig 3B). Hence, *C*_*a*_ *-C*_*i*_ increased linearly over time (*R* ^*2*^ = 0.58, *P <* 0.001; Fig 3C). *W*_*i*_ and mean VPD in the growing season increased linearly (*W*_*i*_: *R* ^*2*^ = 0.56, *P* < 0.001, Fig 4A; VPD: *R* ^*2*^ = 0.14, *P* = 0.009, Fig 4B). *W*_*i*_ increased by 23.5% (approximately 18 μmol·mol^–1^) from 1962 to 2010, which was an increase of 3.20% per 10 μmol·mol^–1^ increase in *C*_*a*_. Mean VPD in the growing season increased by 0.11 kPa; as a result, the observed *W*_*e*_ did not increase significantly from 1962 to 2010 (Fig 4C).

### Relationship between *W*_*i*_ and *W*_*e*_ and environmental parameters

*GST* increased linearly over time (*R* ^*2*^ = 0.51, *n* = 49, *P* < 0.001), and growing season precipitation (*GSP*) was relatively stable (*R* ^*2*^ = 0.01, *n* = 49, *P* = 0.495). *W*_*i*_ increased significantly with increasing *GST* (*R* ^*2*^ = 0.38, *n* = 106, *P* < 0.01) and *C*_a_ (*R* ^*2*^ = 0.55, *n* = 106, *P* < 0.01). *GSP* had no apparent influence on *W*_*i*_ (*R* ^*2*^ = 0.04, *n* = 106, *P* = 0.041).

*W*_*e*_ was positively related to *GSP* (*P* < 0.05) but negatively related to *GST* and *C*_*a*_ (*P* < 0.05). Linear regression showed that *GSP*, *GST*, and *C*_*a*_ jointly explained 45% of the variability in *W*_*e*_ (*W*_*e*_ = 17.671 + 0.046*C*_*a*_ + 0.021*GSP* – 2.332*GST*; *R* ^*2*^ = 0.452, *n* = 106, *P* < 0.001). Partial correlation analysis showed that the relative importance of the three variables was *GSP* (partial correlation coefficient, 0.506) > *GST* (partial correlation coefficient, –0.350) > *C*_*a*_ (partial correlation coefficient, 0.237)_._

## Discussion

### Intrinsic water use efficiency increased in alpine meadows

The average ^*13*^ *Δ*_*veg*_ value (18.10‰) and its intra-annual variation (~2.5‰) were reasonable because they were close to the values of other C_3_ plants on the Tibetan Plateau (average, ~18‰; variation, 4.5‰) [10, 23] and the Mongolian Plateau (average, ~17‰; variation, 1.9‰) [21]. Intra-annual variation of ^*13*^ *Δ*_*veg*_ might have been a result of the spatial differences in *GST* and *GSP*. The observed *C*_*i*_/*C*_*a*_ ratio varied little over the past half century, which indicated a proportional adjustment of *C*_*i*_ in vegetation to the increase in *C*_*a*_. Three theoretical responses of *C*_*i*_ to increasing *C*_*a*_ include (i) the difference between *C*_*a*_ and *C*_*i*_ remains constant when the increase in *C*_*i*_ and *C*_*a*_ is equal, (ii) the *C*_*i*_/*C*_*a*_ ratio remains consistent because *C*_*i*_ and *C*_*a*_ increase proportionally, and (iii) *C*_*i*_ is constant [12]. The first response suggests that *W*_*i*_ remains constant; the second and third responses indicate that *W*_*i*_ increases with increasing *C*_*a*_. Our results demonstrated that, with the increase in *C*_a_ and *GST*, *C*_*i*_ increased in the alpine meadows of the Tibetan Plateau to keep the *C*_*i*_ */C*_*a*_ ratio relatively stable, which supports the second response.

In terms of gas-exchange (Eq 5) the increase in *W*_*i*_ must result from increased assimilation or decreased stomatal conductance. Previous studies, such as the FACE and OTC experiments [24], are consistent with our findings that elevated CO_2_ stimulates photosynthesis and decreases or has no influence on *g*_*s*_. The significant positive correlations between *W*_*i*_ and *GST* and between *W*_*i*_ and *C*_a_ suggested that the rapid increases in *GST* and *C*_a_ had positive effects on photosynthesis. For *GST*, the main reason was that the increase of *GST* can increase leaf temperature, improve Rubisco enzyme activity in chloroplasts, and enhance *A* although the increasing GST would also enhance leaf respiration. Additionally, *g*_*s*_ could be decreased by the increase of *GST* to decrease water loss. For *C*_a_, the major reason was that the increase of *C*_a_ could increase the supply of CO_2_ to leaf mesophyll cells and then improve photosynthesis. In addition, increased grazing pressure and decreased *GSP* could also theoretically explain the increase in *W*_*i*_, but only to a lesser extent considering that these were not major factors during the study period because they were almost unchanged. Hence, under the background of warming and the increase of *C*_a_, the plants tried to adapt to these changes in environment conditions via the physiological adjustment of the WUE.

Previous studies have reconstructed the responses of WUE in trees on the Tibetan Plateau over the past two centuries using Eq (5) [25–26] and found that *W*_*i*_ increased in response to elevated *C*_a_ and drought. Our findings for *W*_*i*_ were consistent with previous studies that examined this variable in trees in various ecosystems worldwide and in Tibetan alpine meadows (Table 1). These findings suggest that the long-term responses of *W*_*i*_ to increasing *C*_a_ are similar in alpine forests and alpine meadows. However, the long-term response of *W*_*i*_ observed here was 1.5-fold higher than that of alpine grasslands in the Swiss Alps from 1938 to 2006 [12] (Table 1). Possible reasons for this difference include lower air pressure due to higher altitude in our study (4200 m) relative to the Swiss grasslands (2200 m) and difference in species composition.

**Table 1:** Long-term changes in intrinsic water use efficiency (*W*_*i*_) of trees and alpine meadow and in atmospheric CO_2_ concentration (*C*_*a*_).

Systems	Locality	Period	*C*_*a*_ increase (μmol·mol^–1^)	Increase in *W*_*i*_ (%)		References
				Total	Per 10 μmol·mol^–1^ increase of *C*_*a*_	
**Forests**						
*Pinus* sp., *Picea sitchensis*, *Quercus lobata*, *Fitzroya cupressoides*, *Juniperus phoenicea*	Western North America and Chile (*Fitzroya*)	1800–1990	72	5–45	0.70–6.25	[36]
*Larix* sp., *Pinus* sp., *Picea* sp.	Northern Eurasia	1861–1990	67	19.2±0.9	2.87±0.13	[36]
*Fagus sylvatica* (Coppice with standards)	Northeastern France	1850–1990	69	23	3.33	[37]
*Fagus sylvatica* (High forest)	Northeastern France	1850–1990	69	44	6.38	[37]
*Sabina przewalskii*	Tibet, China	1850–2000	83	23.6	2.84	[25]
*Picea crassifolia*	Tibet, China	1850–2000	83	35.5	4.28	[25]
*Picea crassifolia*	Tibet, China	1890–2002	79	34	4.30	[26]
**Grasslands**						
Alpine meadow	Switzerland	1938–2006	81	17.8	2.20	[12]
Alpine meadow	Tibet, China	1962–2010	73	23.5	3.20	This study

A plant community consisting solely of C_3_ species is an important assumption of the use of *δ* ^*13*^ *C*_veg_ to estimate *C*_*i*_ */C*_*a*_ and *W*_*i*_. In general, C_4_ plants are more abundant in areas with mean monthly growing season temperature above 22°C and precipitation above 25 mm [27–29], conditions that do not occur above 3500 m on the Tibetan Plateau [28–30]. Our investigation of plant composition validated the assumption of C_3_ species only in our study area; thus, *W*_*i*_ values estimated from wool were reliable for this region. Another potentially influencing factor of *δ* ^13^ *C*_veg_ could be changes in species composition over time. Unfortunately, we are not aware of any long-term vegetation analyses in our study region or similar ones. However, a short term simulated warming experiment in alpine meadows in Tibet [31] revealed no significant changes in species composition and data from the Damxung Grassland Research Station also showed only minor variation in the relative aboveground biomass of Poaceae (mean, 34.2%; SD, 12%) and Cyperaceae (mean, 30.4%; SD, 9.1%) during 2005–2011, a period of significant warming and *C*_*a*_ increase (Yongtao He, unpublished data). We would therefore conclude that the potential effect of species compositional changes should be minor.

### Effective water use efficiency remained stable

Alpine meadows on the Tibetan Plateau have made physiological adjustments to increase *WUE* under climate warming and elevated *C*_a_, but, unexpectedly, *W*_*e*_ remained relatively stable over the past half century (Fig 4C). Warming and drying trends of the atmosphere as shown by the increase of VPD and the results of Xie and others [32] hindered the improvement of plant WUE. Our assumption that the increase in NPP depended on the increase of WUE was not supported by the results. Plant *W*_*e*_ and available soil water [33] determine the NPP of alpine meadows. Based on the model results of Piao and others [2] and Chen and others [34], the NPP in this region increased during our study period. In this case, a major reason for the increase of NPP may be the increase of water available to the plants. Although *GSP* did not change during 1962–2010, the amount of annual precipitation increased in Damxung County (*R* ^*2*^ = 0.16, *P* = 0.005) and in the whole Tibetan Plateau (*R* ^*2*^ = 0.24, *P* < 0.001) [2]. These results suggest that winter precipitation may be an important water resource in our study area. Similar findings have been reported in the Mongolia grasslands, where the water from winter half-year precipitation contributed 15–45% of the total water uptake by plants [35].

## Supporting Information

S1 Dataset The values of stable carbon isotope, WUE and related climate data in the study. (XLSX)
